# Nitric Oxide Mitigates Salt Stress by Regulating Levels of Osmolytes and Antioxidant Enzymes in Chickpea

**DOI:** 10.3389/fpls.2016.00347

**Published:** 2016-03-31

**Authors:** Parvaiz Ahmad, Arafat A. Abdel Latef, Abeer Hashem, Elsayed F. Abd_Allah, Salih Gucel, Lam-Son P. Tran

**Affiliations:** ^1^Department of Botany, Sri Pratap CollegeSrinagar, India; ^2^Botany Department, Faculty of Science, South Valley UniversityQena, Egypt; ^3^Biology Department, College of Applied Medical Sciences, Taif UniversityTurabah, Saudi Arabia; ^4^Mycology and Plant Disease Survey Department, Plant Pathology Research Institute, Agriculture Research CenterGiza, Egypt; ^5^Botany and Microbiology Department, College of Science, King Saud UniversityRiyadh, Saudi Arabia; ^6^Plant Production Department, College of Food and Agricultural Sciences, King Saud UniversityRiyadh, Saudi Arabia; ^7^Centre for Environmental Research, Near East UniversityNicosia, Cyprus; ^8^Plant Abiotic Stress Research Group & Faculty of Applied Sciences, Ton Duc Thang UniversityHo Chi Minh City, Vietnam; ^9^Signaling Pathway Research Unit, RIKEN Center for Sustainable Resource ScienceYokohama, Japan

**Keywords:** antioxidant enzymes, chickpea, gene expression, nitric oxide, osmolytes, salt stress

## Abstract

This work was designed to evaluate whether external application of nitric oxide (NO) in the form of its donor *S*-nitroso-*N*-acetylpenicillamine (SNAP) could mitigate the deleterious effects of NaCl stress on chickpea (*Cicer arietinum* L.) plants. SNAP (50 μM) was applied to chickpea plants grown under non-saline and saline conditions (50 and 100 mM NaCl). Salt stress inhibited growth and biomass yield, leaf relative water content (LRWC) and chlorophyll content of chickpea plants. High salinity increased electrolyte leakage, carotenoid content and the levels of osmolytes (proline, glycine betaine, soluble proteins and soluble sugars), hydrogen peroxide (H_2_O_2_) and malondialdehyde (MDA), as well as the activities of antioxidant enzymes, such as superoxide dismutase (SOD), catalase (CAT), ascorbate peroxidase (APX), and glutathione reductase in chickpea plants. Expression of the representative *SOD*, *CAT* and *APX* genes examined was also up-regulated in chickpea plants by salt stress. On the other hand, exogenous application of NO to salinized plants enhanced the growth parameters, LRWC, photosynthetic pigment production and levels of osmolytes, as well as the activities of examined antioxidant enzymes which is correlated with up-regulation of the examined *SOD*, *CAT* and *APX* genes, in comparison with plants treated with NaCl only. Furthermore, electrolyte leakage, H_2_O_2_ and MDA contents showed decline in salt-stressed plants supplemented with NO as compared with those in NaCl-treated plants alone. Thus, the exogenous application of NO protected chickpea plants against salt stress-induced oxidative damage by enhancing the biosyntheses of antioxidant enzymes, thereby improving plant growth under saline stress. Taken together, our results demonstrate that NO has capability to mitigate the adverse effects of high salinity on chickpea plants by improving LRWC, photosynthetic pigment biosyntheses, osmolyte accumulation and antioxidative defense system.

## Introduction

Sodium chloride (NaCl) is the prevailing salt in the soil, and the higher concentration of this salt provokes two primary effects on plants, namely the osmotic and ionic effects, of which the osmotic stress minimizes the ability of plants to take up water and minerals (Khan et al., 2012). Furthermore, excessive accumulation of Na^+^ in the cytosol causes toxic effects on cell membranes, leading to electrolyte leakage, as well as constrains the metabolic processes in the cytosol, which ultimately reduce the physiological and biochemical activities (Ahmad, 2010; Ahmad et al., 2014; Hashem et al., 2014). Higher salt concentrations as well as prolonged exposure to NaCl stress cause oxidative stress in plants (Ahmad et al., 2012b; Rasool et al., 2013). Salt and osmotic stresses produce reactive oxygen species (ROS) that cause oxidative stress in plants (Ahmad et al., 2012b; Abdel Latef and Chaoxing, 2014). To deal with the adverse impacts of oxidative stress, plants are furnished with well-regulated antioxidant machinery that can protect biomolecules from further damages caused by the stress (Rasool et al., 2013). The ROS scavenging enzymes involve superoxide dismutase (SOD), catalase (CAT), ascorbate peroxidase (APX) and glutathione reductase (GR) (Evelin and Kapoor, 2014), which exist in different cellular compartments as isoenzymes especially in chloroplasts and mitochondria (Apel and Hirt, 2004; Ahmad et al., 2008, 2010). Accumulation of osmolytes, such as proline, glycine betaine (GB), soluble proteins and soluble sugars, is another strategy to beat osmotic stress provoked by salinity (Khan et al., 2012; Abdel Latef and Chaoxing, 2014).

Nitric oxide (NO) is an important endogenous plant bioactive signaling molecule that has a key function in various processes of plant growth and development, including seed dormancy, seed germination, primary and lateral root growth, floral transition, flowering, pollen tube growth regulation, fruit ripening, gravitropism, stomatal movements, photosynthesis, mitochondrial functionality, senescence, plant metabolism and cell death, as well as stress responses (Siddiqui et al., 2011; Manai et al., 2014; Mostofa et al., 2015). NO plays a pivotal role in stress tolerance exerted by oxidative stress (Siddiqui et al., 2011; Ahmad et al., 2012a). In the past few years, research on function of NO in salt stress tolerance has obtained much interest (Yang et al., 2011; Mostofa et al., 2015). However, the information available is sometimes contradictory, depending on the plant species, severity and duration of the salinity treatments (Begara-Morales et al., 2014; Manai et al., 2014).

Grain legumes belonging to Fabaceae family are rich in proteins and proved to be very important components of human diet (Jukanti et al., 2012). Among the grain legumes, chickpea (*Cicer arietinum* L.) is a very popular crop around the globe because it can supply a rich source of proteins, fats and carbohydrates for humans and animals (Rasool et al., 2015). Chickpea is differentiated as “Kabuli-type” and “desi-type” on the basis of size and color of the seeds. Kabuli-type seeds are bold with thin and white seed coat, while those of desi-type are small in size with thicker seed coat and having color ranging from brown to yellow (Khan et al., 1995; Rasool et al., 2015).

Chickpea normally grows under rainfed and irrigated conditions (Rasool et al., 2015). The soil used for the cultivation of chickpea should be free from high salinity as this crop is very sensitive to salinity stress. For instance, Kotula et al. (2015) reported that exposure of chickpea genotypes to 50 mM of NaCl stress decreased the plant growth and yield. Thus, steps are to be taken to enhance the salinity tolerance of chickpea genotypes in order to grow them on natural saline soil. Considering the vital role of NO in plant stress responses and management, the present study was designed to evaluate the influences of exogenous NO in mitigating high salinity-induced negative effects on growth and physiological attributes of chickpea plants. Additionally, the effects of exogenous NO treatment on accumulation of key osmolytes, activities of antioxidant enzymes and expression of representative antioxidant enzyme-encoding genes were examined in salt-stressed chickpea plants.

## Materials and Methods

### Plant Materials and Treatments

Seeds of chickpea (*Cicer arietinum* L.) were planted in earthen pots containing peat, perlite and sand (1:1:1, v/v/v) under glass house. Thinning was carried out to accommodate one plant per pot after 4 days of germination. Subsequently, the seedlings were grown for three more weeks under average day/night temperature of 24°C/15°C. Thereafter, 25-day-old-plants were treated with:

(1) Nutrient solution alone (control) (T0): 0 mM NaCl + 0 μM SNAP
(2) NO alone (T1): 0 mM NaCl + 50 μM SNAP
(3) Salt stress alone (T2): 50 mM NaCl + 0 μM SNAP
(4) Salt stress and NO (T3): 50 mM NaCl + 50 μM SNAP
(5) Salt stress alone (T4): 100 mM NaCl + 0 μM SNAP
(6) Salt stress and NO (T5): 100 mM NaCl + 50 μM SNAP

NaCl and NO were given to pots dissolved in nutrient solution every week from the first day of treatment (i.e., 25-day-old plants) up to day 45th (70-day-old plants). Collection of samples was done after 45 days of treatment. The nutrient solution is made up of (mg l^-1^): N 270, P 31, K 234, Ca 200, S 64, Mg 48, Fe 2.8, Mn 0.5, Cu 0.02, Zn 0.05, and Mo 0.01. 0.1 M KOH was used to adjust the pH of nutrient solution to 6.5. The experiment was laid out in randomized block design with five replicates in each treatment, and each replicate comprised five plants.

### Determination of Growth Parameters

Shoot and root lengths were measured using measuring scale. Shoot dry weight (DW) was measured after the plant samples were dried at 70°C for 72 h.

### Estimation of Leaf Relative Water Content and Electrolyte Leakage

Leaf relative water content (LRWC) was assayed using the method of Yamasaki and Dillenburg (1999). RWC was calculated using the following formula:

$$RWC\left(\%\right)=\frac{Fresh\,\;weight-Dry\,\;weight}{Turgid\,\;weight-Dry\,\;weight}\times 100.$$

Electrolyte leakage was estimated as described previously (Dionisio-Sese and Tobita, 1998). First, the electrical conductivity (EC_a_) of 20 leaf disks submerged in deionized water was measured. Subsequently, the test tubes containing the leaf discs were incubated in water bath at temperature 50°C–60°C for 25 min, and the electrical conductivity (EC_b_) of the samples was determined. Finally, these test tubes were boiled at 100°C for 10 min, and then the electrical conductivity (EC_c_) was measured. The electrolytic leakage was calculated using the following formula:

$$Electrolyte\,\;\;leakage\,\;(\%)=\frac{({EC}_{b}-{EC}_{a})\times 100}{{EC}_{c}}$$

### Determination of the Contents of Photosynthetic Pigments

The method of Hiscox and Israelstam (1979) was used for the estimation of photosynthetic pigments using dimethyl sulphoxide (DMSO) as the extraction reagent. The absorbances at 480, 510, 645, and 663 nm were recorded by spectrophotometer (Beckman 640 D, USA), with DMSO being used as a blank.

### Estimation of the Contents of Proline, GB, Soluble Proteins, and Soluble Sugars

Estimation of proline contents in fresh leaf samples was carried out as previously described by Bates et al. (1973). The absorbance was taken at 520 nm using a spectrophotometer (Beckman 640 D, USA), with toluene serving as a blank. GB contents in fresh leaf samples were measured according to Grieve and Grattan (1983). The absorbance was spectrophotometerically determined at 365 nm. GB (50–200 mg ml^-1^) prepared in 1N H_2_SO_4_ was used as control. Soluble protein content and soluble sugar content in fresh leaves were determined by the methods of Bradford (1976) and (Dey, 1990), respectively.

### Determination of Hydrogen Peroxide (H_2_O_2_) Content and Lipid Peroxidation

H_2_O_2_ contents were estimated in dried leaf samples using the method of Velikova et al. (2000). Lipid peroxidation was assayed by quantifying the malondialdehyde (MDA) contents in fresh leaf samples using the method of Rao and Sresty (2000).

### Enzyme Assays

The fresh leaf samples (0.5 g per sample) were homogenized in presence of phosphate buffer (0.1 M, pH 7.5) and ethylenediaminetetraacetic acid (EDTA, 0.5 mM). Subsequently, the samples were centrifuged at 12,000 ×*g* for 10 min at 4°C after the filtration. The supernatants collected served as sources for determination of SOD (EC 1.15.1.1), CAT (EC 1.11.1.6) and GR (EC 1.6.4.2) activities. For determination of APX (EC 1.11.1.11) activity, leaf samples were separately grounded in a homogenizing medium containing phosphate buffer (0.1 M, pH 7.5), 0.5 mM EDTA and 2 mM ascorbic acid (AsA).

Superoxide dismutase activity was determined by photoreduction of nitro blue tetrazolium (NBT) (Bayer and Fridovich, 1987). The absorbance was recorded at 560 nm using a spectrophotometer (Beckman 640 D, USA). One unit of SOD is the amount of protein regulating 50% photoreduction of NBT. The activity of SOD was expressed as enzyme unit (EU) mg^-1^ protein. For the estimation of CAT activity, the procedure of Aebi (1984) was employed. The absorbance was read at 240 nm using a spectrophotometer (Beckman 640 D, USA), and EU mg^-1^ protein expresses the CAT activity. APX activity was assayed using the method of Nakano and Asada (1981). The absorbance was spectrophotometerically determined at 290 nm. One unit of APX is the amount of protein used to decompose 1 μmol of substrate min^-1^ at 25°C, which was shown as EU mg^-1^ protein to express the APX activity. The method of Foyer and Halliwell (1976) was exerted for the determination of GR activity. The optical density (OD) was recorded at 340 nm using a spectrophotometer (Beckman 640 D, USA). GR activity was expressed as μmol NADPH oxidized min^-1^ (EU mg^-1^ protein).

### Expression of *SOD*, *CAT*, and *APX* Genes

Total RNA was extracted from leaf samples using Trizol (Promega) according to the protocol of manufacturer. RNA samples were treated with DNase I (Promega) before their absorbance was read at 260 and 280 nm to determine RNA concentration and purity. The first-strand cDNA was synthesized from 5 μg RNA template using GoScript^TM^ Reverse Transcription System (Promega) according to the manufacturer’s instruction, with oligo (dT) 18 as a primer. Real-time quantitative PCR (RT-qPCR) was carried out using the QuantiTect SYBR Green PCR Kit (Qiagen) and Light Cycler (Model 480, Roche) with gene-specific primers designed for *SOD* (F: 5′-ACATTTGCTACCTCTCCCTCACCT-3′; R: 3′-TCGGGTAAGACATCGTCGGTATGT-5′), *CAT* (F: 5′-GGCGGTACGTTTACGATTTACGCT-3′; R: 3′- ACCTATCACGGGTCAGCACGATTT-5′) and *APX* (F: 5′-AAACCCAAGCTCAGAGAGCCTCAT-3′; R: 3′-TACTTCACGGTGCTTCTTGGTGGA-5′).

To standardize the results, the relative abundance of *β-Actin* (AB047313) reference gene (F: 5′-TGATGGTGTCAGCCACACT-3; R: 5′TGGTCTTGGCAGTCTCCATT-3) was also determined, which was then defined as 100 relative expression units (REU) and used as the internal standard. The expression level of a gene corresponds to the ratio of the copy number of cDNA of the studied gene to the copy number of *β-Actin* gene multiplied by 100 REU. These representative *SOD*, *CAT* and *APX* genes were selected as they give the highest expression levels when compared with other homologous versions (Hu et al., 2011).

### Statistical Analysis

Duncan’s Multiple Range Test (DMRT) was carried out using the One-way Analysis of Variance (ANOVA). The values obtained were the means ± standard errors (SEs) of five replicates in each group. *P*-values ≤0.05 were considered as significant.

## Results

### NO Improves Growth and Biomass Yield under NaCl Stress

Exposure of chickpea plants to salinity stress resulted in a drastic decline in growth parameters expressed as shoot length, root length and shoot DW compared with untreated control (**Table 1**). The shoot length decreased by 18.52 and 40.58% at T2 (50 mM NaCl + 0 μM SNAP) and T4 (100 mM NaCl + 0 μM SNAP) treatments, respectively, relative to T0 control (0 mM NaCl + 0 μM SNAP). Application of NO in presence of NaCl showed an increment by 11.88% at T3 (50 mM NaCl + 50 μM SNAP) and 20.50% at T5 (100 mM NaCl + 50 μM SNAP) treatments as compared with T2 and T4, respectively. Root length was also negatively affected by NaCl stress, as T2 and T4 treatments decreased the root length by 36.90 and 59.80%, respectively, relative to T0 control. Supply of NO to NaCl-treated plants at T3 and T5 treatments boosted the root length by 12.98 and 17.85% as compared with T2 and T4, respectively. A decrease by 30.48 and 51.66% at T2 and T4, respectively, was also observed in shoot DW as compared with T0 control. However, supplementation of NO to salt-stressed plants improved the shoot DW, and the increase was 20.21% at T3 and 26.69% at T5 over T2 and T4 treatments, respectively. No significant change was observed in the examined parameters at T1 (0 mM NaCl + 50 μM SNAP) treatment compared with T0 control (**Table 1**).

**Table 1:** Effects of NO on growth and biomass yield of chickpea plants under salt stress.

Treatments	Shoot length (cm plant^-1^)	Root length (cm plant^-1^)	Shoot DW (g plant^-1^)
T0	40.71 ± 2.16^a^	22.71 ± 1.37^a^	14.73 ± 1.07^a^
T1	42.23 ± 2.20^a^	23.55 ± 1.40^a^	15.76 ± 1.11^a^
T2	33.17 ± 1.34^c^	14.33 ± 1.05^c^	10.24 ± 0.91^c^
T3	37.11 ± 1.70^b^	16.19 ± 1.12^b^	12.31 ± 0.98^b^
T4	24.19 ± 1.13^e^	9.13 ± 0.82^e^	7.12 ± 0.77^e^
T5	29.15 ± 1.27^d^	10.76 ± 0.95^d^	9.02 ± 0.85^d^

*Data presented are the means ± SEs (n = 5). Different letters next to the number indicate significant difference (P ≤ 0.05). T0 (control) = 0 mM NaCl + 0 μM SNAP; T1 = 0 mM NaCl + 50 μM SNAP; T2 = 50 mM NaCl + 0 μM SNAP; T3 = 50 mM NaCl + 50 μM SNAP; T4 = 100 mM NaCl + 0 μM SNAP; T5 = 100 mM NaCl + 50 μM SNAP. DW, dry weight.*

### Effects of NaCl and NO on LRWC and Electrolyte Leakage

LRWC was reduced by 21.54% at T2 (50 mM NaCl + 0 μM SNAP), and the maximum decrease (46.93%) in LRWC was recorded at T4 (100 mM NaCl + 0 μM SNAP) relative to T0 (0 mM NaCl + 0 μM SNAP) control (**Table 2**). The decrease in LRWC of salt-stressed plants was alleviated by exogenous application of NO, resulting in an enhancement in LRWC of 15.72 and 33.62% at T3 (50 mM NaCl + 50 μM SNAP) and T5 (100 mM NaCl + 50 μM SNAP), respectively, as compared with plants treated with NaCl only (T2 and T4, respectively). On the other hand, electrolyte leakage of chickpea plants increased by salt stress, and maximum elevation of 4.60-fold was recorded at T4 treatment compared with T0 control (**Table 2**). Exogenous application of NO reduced the electrolyte leakage in salt-stressed plants by 27.08% at T3 and 21.33% at T5 in comparison with NaCl-treated plants alone (T2 and T4, respectively). NO treatment alone (T1; 0 mM NaCl + 50 μM SNAP) had insignificant effect on LRWC and electrolyte leakage of chickpea plants as compared with T0 control (**Table 2**).

**Table 2:** Effects of NO on leaf relative water content (LRWC), electrolyte leakage, and the contents of proline, glycine betaine (GB), total soluble proteins and total soluble sugars in leaves of chickpea plants under salt stress.

Treatments	LRWC (%)	Electrolyte leakage (%)	Proline (μg g^-1^ FW)	GB (μmol g^-1^ FW)	Total soluble proteins (mg g^-1^ FW)	Total soluble sugars (mg g^-1^ FW)
T0	85.13 ± 2.57^a^	14.17 ± 1.05^d^	27.10 ± 1.69^e^	2.40 ± 0.18^e^	18.31 ± 0.89^e^	6.20 ± 0.51^e^
T1	87.10 ± 2.63^a^	13.21 ± 0.99^d^	30.75 ± 1.79^e^	2.70 ± 0.15^e^	21.32 ± 1.16^cd^	6.40 ± 0.54^e^
T2	66.79 ± 2.24^bc^	25.66 ± 1.74^c^	75.26 ± 2.23^d^	10.71 ± 0.96^d^	24.52 ± 1.26^d^	7.71 ± 0.68^d^
T3	77.29 ± 2.48^ab^	18.71 ± 121^cd^	88.11 ± 3.16^c^	15.35 ± 1.12^c^	30.14 ± 1.44^c^	7.92 ± 0.75^c^
T4	45.18 ± 2.57^d^	65.12 ± 2.24^a^	105.29 ± 3.75^b^	19.22 ± 1.22^b^	36.77 ± 1.72^b^	8.53 ± 0.83^b^
T5	60.37 ± 2.14^c^	51.23 ± 2.06^b^	130.77 ± 3.95^a^	24.72 ± 1.41^a^	39.81 ± 1.95^a^	9.07 ± 0.92^a^

*Data presented are the means ± SEs (n = 5). Different letters next to the number indicate significant difference (P ≤ 0.05). T0 (control) = 0 mM NaCl + 0 μM SNAP; T1 = 0 mM NaCl + 50 μM SNAP; T2 = 50 mM NaCl + 0 μM SNAP; T3 = 50 mM NaCl + 50 μM SNAP; T4 = 100 mM NaCl + 0 μM SNAP; T5 = 100 mM NaCl + 50 μM SNAP. FW, fresh weight.*

### NO Mitigates the Effects of NaCl Stress on Photosynthetic Pigment Biosyntheses

**FIGURE 1:**
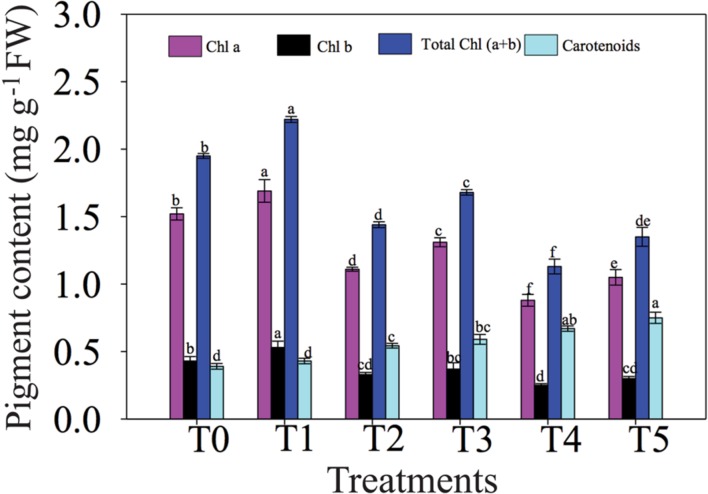
**Effects of NO on chlorophyll (Chl) and carotenoid contents in leaves of chickpea plants under salt stress.** Data presented are the means ± SEs (*n* = 5). Different letters indicate significant difference (*P* ≤ 0.05) among the treatments. T0 (control) = 0 mM NaCl + 0 μM SNAP; T1 = 0 mM NaCl + 50 μM SNAP; T2 = 50 mM NaCl + 0 μM SNAP; T3 = 50 mM NaCl + 50 μM SNAP; T4 = 100 mM NaCl + 0 μM SNAP; T5 = 100 mM NaCl + 50 μM SNAP. FW, fresh weight.

The Chl a, Chl b and total Chl contents significantly decreased by salt stress and the percent reduction in these parameters was nearly equal (≈ 42%) at T4 (100 mM NaCl + 0 μM SNAP) treatment in comparison with T0 (0 mM NaCl + 0 μM SNAP) control. The levels of carotenoids sharply increased with increasing NaCl stress intensity, and the maximum increase (71.79%) was recorded at the T4 treatment over the T0 control. Supplementation of NO elevated the contents of Chl a, Chl b and total Chl by 19.32, 20, and 19.47%, respectively, and that of carotenoids by 11.94% at T5 (100 mM NaCl + 50 μM SNAP) in NO + NaCl-treated plants over plants treated with NaCl alone (T4) (**Figure 1**). Exogenous NO treatment showed a positive effect on Chl biosynthesis in chickpea plants under normal conditions as control plants treated with NO (T1; 0 mM NaCl + 50 μM SNAP) showed a significant increase in Chl contents relative to untreated T0 control (**Figure 1**).

### Effects of NaCl and NO on the Contents of Proline, GB, Total Soluble Proteins and Total Soluble Sugars

NaCl triggered the induction of proline biosynthesis by 2.78-fold and 3.89-fold at T2 (50 mM NaCl + 0 μM SNAP) and T4 (100 mM NaCl + 0 μM SNAP) treatments, respectively, versus T0 (0 mM NaCl + 0 μM SNAP) control (**Table 2**). Exogenous application of NO induced the proline biosynthesis by 17.07% at T3 (50 mM NaCl + 50 μM SNAP) and 24.20% at T5 (100 mM NaCl + 50 μM SNAP) treatments over T2 and T4, respectively (**Table 2**). With regard to GB, it markedly accumulated in chickpea plants treated with NaCl alone and in combination with NO (**Table 2**). At concentrations T2 and T4, the accumulation of GB was 4.46- and 8.01-fold, respectively, as compared with T0 control. Supplementation of NO enhanced the accumulation of GB by 43.32 and 28.62% at T3 and T5 treatments, respectively, in comparison with T2 and T4, respectively (**Table 2**).

As for the soluble proteins, their total content increased by 33.91 and 100.81% at T2 and T4 treatments relative to T0 control (**Table 2**). An enhancement by 22.92% (T3) and 8.27% (T5) in soluble protein content was also observed in plants treated with both NaCl and NO, as compared with plants treated with NaCl alone (T2 and T4, respectively) (**Table 2**). In addition, chickpea seedlings treated with T2 and T4 showed elevated soluble sugar content by 24.36 and 37.58%, respectively, over the T0 control (**Table 2**). Application of NO further increased the soluble sugar content in T3- and T5-treated plants relative to T0-plants; however, in comparison with their respective T2- and T4-treated plants, the observed increment was not large, with only 2.72 and 6.33% (**Table 2**). We noticed that NO treatment (T1) alone resulted in a significant change (16.43%) in soluble protein content only in comparison with T0 control (**Table 2**).

### Effects of NaCl and NO on H_2_O_2_ and MDA Contents

**FIGURE 2:**
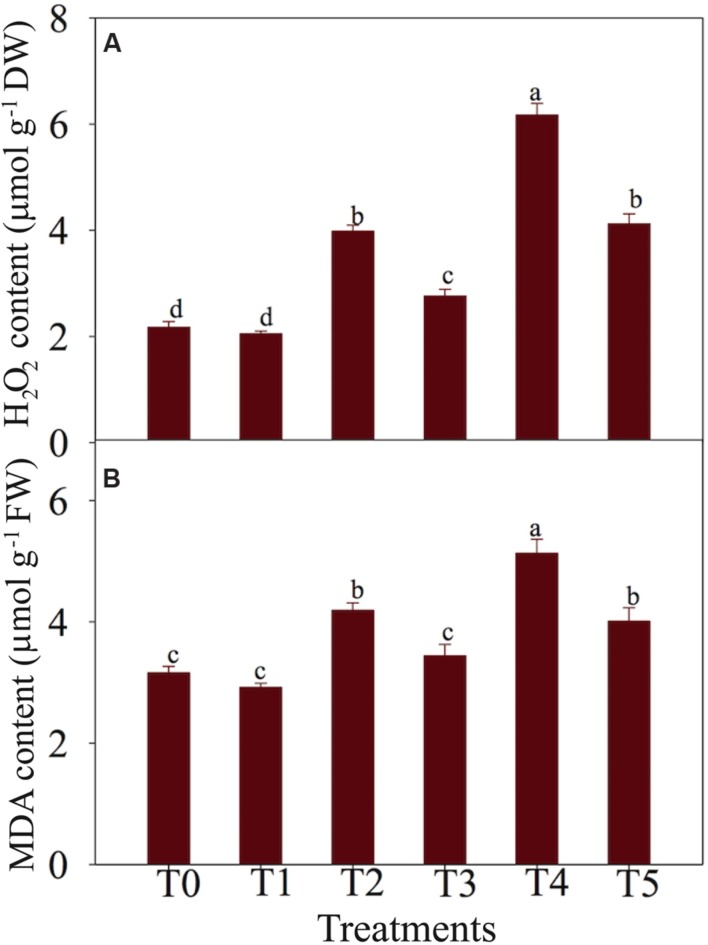
**Effects of NO on (A) hydrogen peroxide (H_2_O_2_) content and (B) malondialdehyde (MDA) content in leaves of chickpea plants under salt stress.** Data presented are the means ± SEs (*n* = 5). Different letters indicate significant difference (*P* ≤ 0.05) among the treatments. T0 (control) = 0 mM NaCl + 0 μM SNAP; T1 = 0 mM NaCl + 50 μM SNAP; T2 = 50 mM NaCl + 0 μM SNAP; T3 = 50 mM NaCl + 50 μM SNAP; T4 = 100 mM NaCl + 0 μM SNAP; T5 = 100 mM NaCl + 50 μM SNAP. DW, dry weight; FW, fresh weight.

The results regarding the impacts of NaCl and NO on H_2_O_2_ and MDA contents in chickpea plants are depicted in **Figures 2A,B**. Increase in H_2_O_2_ contents was observed with the raise of NaCl dose applied to chickpea plants (**Figure 2A**). H_2_O_2_ content increased by 83.41 and 184.33% at T2 (50 mM NaCl + 0 μM SNAP) and T4 (100 mM NaCl + 0 μM SNAP), respectively, versus T0 (0 mM NaCl + 0 μM SNAP) control. Supplementation of exogenous NO to NaCl-stressed plants decreased H_2_O_2_ content by 30.65% and 33.23% in T3 (50 mM NaCl + 50 μM SNAP) and T5 (100 mM NaCl + 50 μM SNAP) treatments, respectively, as compared with plants treated with NaCl alone (T2 and T4, respectively) (**Figure 2A**). As for MDA, its content markedly accumulated in salt-stressed chickpea plants in the present study (**Figure 2B**). An increase by 32.59 and 62.34% in MDA content in T2 and T4 treatments, respectively, was recorded as compared with T0 control. Salt-treated plants supplied with NO showed a decrease by17.90% at T3 and 21.83% at T5 treatments relative to their respective T2 and T4 treatments (**Figure 2B**). No significant change in H_2_O_2_ and MDA contents was noted in T1 (0 mM NaCl + 50 μM SNAP)-treated plants versus T0 control (**Figures 2A,B**).

### Effects of NaCl and NO on Antioxidant Enzyme Activities

**FIGURE 3:**
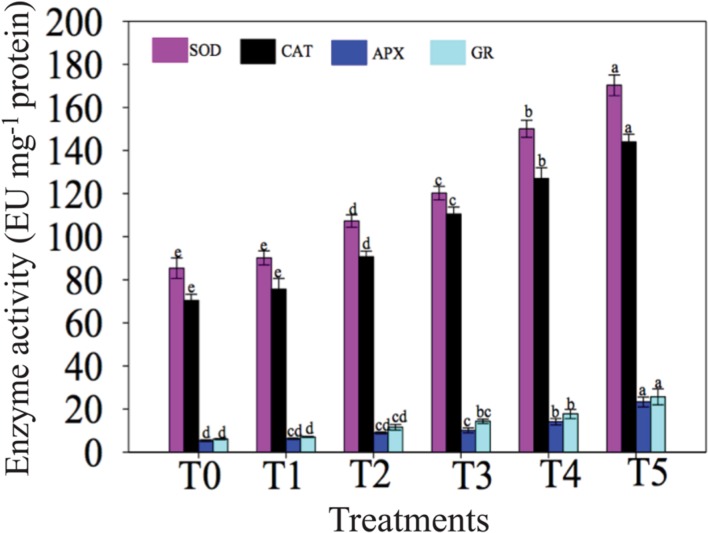
**Effects of NO on activities of superoxide dismutase (SOD), catalase (CAT), ascorbate peroxidase (APX), and glutathione reductase (GR) in leaves of chickpea plants under salt stress.** Data presented are the means ± SEs (*n* = 5). Different letters indicate significant difference (*P* ≤ 0.05) among the treatments. T0 (control) = 0 mM NaCl + 0 μM SNAP; T1 = 0 mM NaCl + 50 μM SNAP; T2 = 50 mM NaCl + 0 μM SNAP; T3 = 50 mM NaCl + 50 μM SNAP; T4 = 100 mM NaCl + 0 μM SNAP; T5 = 100 mM NaCl + 50 μM SNAP. EU, enzyme unit.

The activities of antioxidant enzymes significantly increased in response to NaCl with or without application of exogenous NO (**Figure 3**). Maximum salt stress-induced elevation by 75.83, 80.40, 164.73, and 191.93% in SOD, CAT, APX and GR activities, respectively, was recorded in chickpea plants of T4 (100 mM NaCl + 0 μM SNAP) treatment versus T0 (0 mM NaCl + 0 μM SNAP). Moreover, exogenous application of NO to salt-exposed plants had an additive impact on the activities of antioxidant enzymes. The highest values for SOD, CAT, APX and GR activities were noted in chickpea plants subjected to T5 (100 mM NaCl + 50 μM SNAP) treatment with the increase of 13.44, 13.39, 64.85, and 44.81%, respectively, as compared with plants treated with NaCl alone (T4 treatment). No significant alteration was observed in antioxidant enzyme activities at T1 (0 mM NaCl + 50 μM SNAP) treatment compared with T0 control (**Figure 3**).

### Impacts of NaCl and NO on Transcript Levels of Genes Encoding SOD, APX, and CAT Enzymes

**FIGURE 4:**
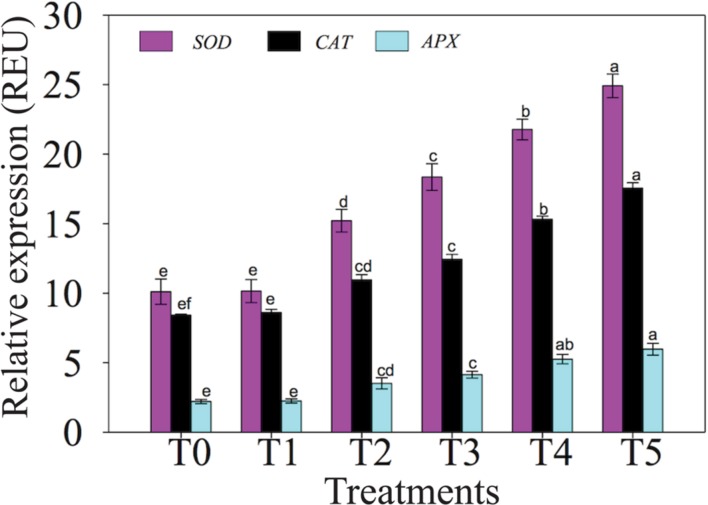
**Effects of NO on expression levels of selected *SOD*, *CAT* and *APX* genes in leaves of chickpea plants under salt stress.** Data presented are the means ± SEs (*n* = 5). Different letters indicate significant difference (*P* ≤ 0.05) among the treatments. T0 (control) = 0 mM NaCl + 0 μM SNAP; T1 = 0 mM NaCl + 50 μM SNAP; T2 = 50 mM NaCl + 0 μM SNAP; T3 = 50 mM NaCl + 50 μM SNAP; T4 = 100 mM NaCl + 0 μM SNAP; T5 = 100 mM NaCl + 50 μM SNAP. REU, relative expression unit.

The expression of SOD, APX and CAT antioxidant enzymes-related genes in leaves of chickpea plants under high salinity in presence and absence of NO is presented in **Figure 4**. Expression of selected genes up-regulated under NaCl stress with or without supplementation of exogenous NO. *SOD, CAT* and *APX* genes showed up-regulation of 2.15-, 1.81-, and 2.38-fold in chickpea plants of T4 (100 mM NaCl + 0 μM SNAP) treatment, respectively, over T0 (0 mM NaCl + 0 μM SNAP) control. Moreover, supplementation of NO to NaCl-treated plants also displayed a remarkable increase in expression level of *SOD* (14.42%), *CAT* (14.63%) and *APX* (13.50%) in T5-treated plants versus T4-treated ones. Insignificant change in expression level of examined genes was recorded in T1 (0 mM NaCl + 50 μM SNAP)-treated chickpea plants in comparison with T0 control (**Figure 4**).

## Discussion

Nitric oxide is an important signaling molecule involved in amelioration of growth and development of plants under various biotic and abiotic stresses (Kausar and Shahbaz, 2013; Liu et al., 2013; Esim and Atici, 2014; Manai et al., 2014). In the present study, salt stress significantly reduced the growth and biomass yield of chickpea plants (**Table 1**), which is in harmony with earlier reports on different crops, such as wheat (*Triticum aestivum*) (Kausar et al., 2013), tomato (*Lycopersicon esculentum*) (Abdel Latef and Chaoxing, 2011), pepper (*Capsicum annuum*) (Abdel Latef and Chaoxing, 2014) and rice (*Oryza sativa*) (Mostofa et al., 2015). Co-application of NO markedly ameliorated shoot length, root length and shoot DW of chickpea plants under high salinity (**Table 1**), which was in agreement with previous findings in many other crops, including wheat (Hasanuzzaman et al., 2011) and rice (Mostofa et al., 2015). Huaifu et al. (2007) reported that supplementation of NO promoted growth of plants exposed to saline conditions. NO can relax the cell wall, act on the phospholipids bilayer, increase membrane fluidness and induce cell enlargement and plant growth (Leshem and Haramaty, 1996). Dong et al. (2014) found that NO application resulted in an improvement in stem and root lengths of cotton (*Gossypium hirsutum*) seedlings under salt stress. They reported that NO is involved in increasing osmotic pressure of the plant cells and improving the cytoplasmic viscosity under high salinity. Zhang et al. (2004) and Wu et al. (2011) working on soybean (*Glycine max*) and maize (*Zea mays*), respectively, demonstrated that application of NO enhanced the plant growth under saline conditions, which might be due to increased activities of antioxidant enzymes. More recently, NO was shown to alleviate the effects of both biotic and abiotic stresses on plants by mediating H_2_O_2_- and salicylic acid-induced mitigation of oxidative damage through the up-regulation of the antioxidant defense (Klessig et al., 2000; Mostofa et al., 2015; Singh et al., 2015).

RWC is adversely affected by imposition of NaCl, which leads to decease in water uptake and injury of root system (Zeng et al., 2011). In the present study, supplementation of NO had a positive impact on LRWC of chickpea plants under salt stress (**Table 2**), which corroborated with previous reports on other plants, such as mustard (*Brassica juncea*) (Zeng et al., 2011) and rice (Habib and Ashraf, 2014). Khan et al. (2012) reported that NO application helped mustard plants retain more water under salt stress. It is still unclear that how NO is able to maintain RWC in stressed plants; however, it has been reported that NO could decrease solute potential, while increasing water potential in plants under osmotic stress (Ke et al., 2013).

The reduction in Chl content of chickpea leaves observed under NaCl stress (**Figure 1**) might be ascribed to the destruction of Chl pigments, decreased Chl syntheses and the vulnerability of the pigment-protein complexes (Rasool et al., 2013). This decrease in Chl content might partially cause a decrease in growth and biomass yield (**Figure 1**; **Table 1**). Carotenoids have been known to play a key role in photosynthetic reaction center in which they are involved in mechanisms regulating photo protection against auto-oxidation (Yang et al., 2013; Gururani et al., 2015). The synthesis of carotenoids was noted to be increased in chickpea under salinity stress (**Figure 1**), perhaps because these compounds act as antioxidants to minimize the oxidative damage induced by NaCl stress. NO was found to provoke the enhancement of photosynthetic pigments in chickpea plants (**Figure 1**), potentially by defending the membrane of the cell organelle containing Chl against salt-induced ion toxicity (Kausar et al., 2013). The enhancement in photosynthetic pigments due to NO application has also been reported in different plant species under salt stress, including wheat (Ruan et al., 2004), tomato (Wu et al., 2010) and rice (Habib et al., 2013).

**FIGURE 5:**
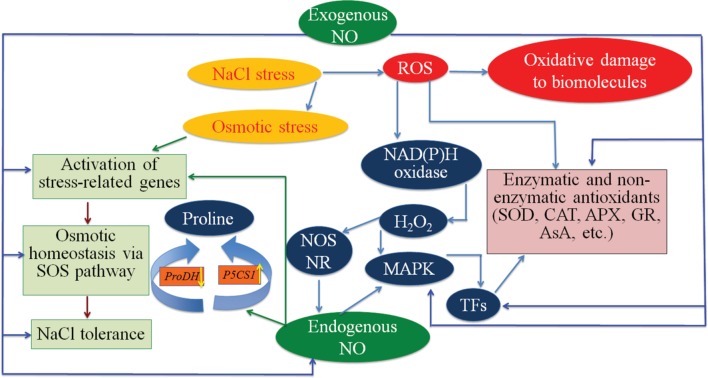
**Potential mechanisms of NaCl stress mitigation by application of exogenous NO.** Excessive NaCl causes osmotic and oxidative stresses in plants. Salt stress induces ABA accumulation, which promotes H_2_O_2_ generation through NAD(P)H oxidase. Stress-induced H_2_O_2_ triggers generation of endogenous NO by activating NR (nitrate reductase) and NOS (nitric oxide synthase)-like enzymes. Exogenous application of NO to plants may enhance the biosynthesis of endogenous NO, as well as that of antioxidant enzymes through MAPK (mitogen-activated protein kinase) and other unknown signaling pathways. Exogenous NO supplementation to plants can also up-regulate genes involved in proline synthesis, such as *P5CS1*, and other stress-related genes responsible for NaCl tolerance, whereas it might down-regulate *ProDH* that is involved in proline catabolism. Exogenous NO treatment may also help balance osmotic homeostasis in plants under salt stress via the SOS (salt overly sensitive) pathway, by increasing plasma membrane H^+^-ATPase activity. APX, ascorbate peroxidase; AsA, ascorbic acid; CAT, catalase; GR, glutathione reductase; H_2_O_2_, hydrogen peroxide; *P5CS1*, *δ1-pyrroline-5-carboxylate synthetase*; *ProDH*, *proline dehydrogenase*; ROS, reactive oxygen species; SOD, superoxide dismutase; TFs, transcription factors.

Salt-stressed chickpea leaves accumulated higher levels of H_2_O_2_ and MDA contents (**Figures 2A,B**), thereby increasing electrolyte leakage (**Table 2**), which might be due to membrane destruction caused by ROS-induced oxidative damage. Exogenous application of NO reduced electrolyte leakage and the levels of MDA and H_2_O_2_ in NaCl-treated chickpea plants (**Figures 2A,B**), which is in agreement with the observations of Zheng et al. (2009) and Khan et al. (2012). Therefore, application of NO could be an effective practice to protect plants against oxidative injury caused by salt stress. Jasid et al. (2008) reported that NO acts as an antioxidant and ROS scavenger, decreasing electrolyte leakage in sorghum (*Sorghum bicolor*). NO stimulates mitogen-activated protein kinase (MAPK) (Neill et al., 2008), which in turn activates transcription factors for induction of stress-related genes (**Figure 5**). Another study by Huaifu et al. (2007) also suggested that NO possesses the ability of restoring and defending the cell membrane to mitigate the damage in the cell membrane system by reducing the membrane permeability and membrane lipid peroxidation, thereby preventing electrolyte leakage.

To overcome the negative impacts of salt stress-induced osmotic stress, plants produce higher levels of osmolytes in the cytosol and other organelles (Abdel Latef and Miransari, 2014). In the present study, a similar accumulation trend of proline, GB, total soluble proteins and total soluble sugars was recorded in chickpea leaves under NaCl stress (**Table 2**). Increased accumulation of total soluble sugars and total soluble proteins in response to saline stress was reported by Liu et al. (2016) in *Nitraria tangutorum*. Proline and GB were also reported to accumulate under salt stress in *B. juncea* (Siddiqui et al., 2008; Khan et al., 2012), linseed (*Linum usitatissimum*) (Khan et al., 2010) and mulberry (*Morus alba*) (Ahmad et al., 2014). Proline and GB are important osmolytes that help in cell osmoregulation under salt stress (Ahmad et al., 2010, 2015). Proline is also reported to protect photosynthetic machinery, and act as energy storage under NaCl stress (Khan et al., 2013; Reddy et al., 2015). Proline has the ability to scavenge ROS and shield the cell from the oxidative damage (Ahmad et al., 2010; Khan et al., 2010; Jogaiah et al., 2013). Verdoy et al. (2006) have reported that proline accumulation enhanced the N_2_ fixation in *Medicago truncatula* plants under salt stress. GB has been reported to inhibit accumulation of ROS, protect photosynthetic machinery and activate stress-related genes (Chen and Murata, 2008). GB is also known to maintain the protein structures from damage induced by abiotic stresses (Sakamoto and Murata, 2002). Soluble proteins play a main role in osmotic adjustment under NaCl stress and can provide storage form of nitrogen (Singh et al., 1987). Increase in soluble protein content under stress may be the result of enhanced synthesis of specific stress-related proteins (Doganlar et al., 2010). Soluble sugars act as important osmolytes to maintain the cell homeostasis (Gupta and Kaur, 2005). Change in total soluble sugars under NaCl stress also involves changes in CO_2_ assimilation, enzyme activities and expression of certain genes (Gibson, 2005; Gupta and Kaur, 2005). Thus, application of NO to salt-stressed chickpea plants provoked a remarkable increase in levels of total soluble proteins, proline, GB and soluble sugars, perhaps to provide a better protection to plants exposed to stress. The protective nature of the osmolytes under NO treatment corroborates with the findings of Hayat et al. (2012), Khan et al. (2012), Kausar et al. (2013), and Dong et al. (2014) in various plants. Exogenous application of NO has also been known to induce the *P5CS1* gene encoding δ1-pyrroline-5-carboxylate synthetase, a key enzyme involved in the proline synthesis (Zhang et al., 2008; Rejeb et al., 2014) (**Figure 5**). Noticeable accumulation of proline, GB, total soluble proteins and total soluble sugars due to NO application might enhance salt tolerance of cells through osmotic regulation. As a consequence, the increased osmotic pressure in the cells increased water uptake, and subsequently RWC, plant growth and biomass yield (**Tables 1** and **2**).

Salt stress induces the generation of huge amount of ROS, leading to the abnormalities at cellular level due to oxidation of proteins, lipids and nucleic acids (Schutzendubell and Polle, 2002; Ahmad et al., 2008, 2010; Hayat et al., 2012) (**Figure 5**). However, plants are capable to deal with such stressful conditions through increasing synthesis of antioxidant metabolites, including proline, and antioxidant enzymes, such as SOD, CAT, APX and GR (Schutzendubell and Polle, 2002; Ahmad et al., 2008, 2010; Hayat et al., 2012). In present study, the increase in the activities of SOD, CAT, APX and GR, as well as of proline content in chickpea plants due to salinity was observed (**Figure 3**; **Table 2**). Our results are supported by the observations reported by Hayat et al. (2012) in *Solanum lycopersicum*, Kausar et al. (2013) in *T. aestivum* and Manai et al. (2014) in *S. lycopersicum*. Furthermore, co-application of NO with NaCl markedly increased the activities of SOD, CAT, APX and GR in chickpea plants (**Figure 3**), which is in harmony with previous findings reported in mustard (Khan et al., 2012), tomato (Hayat et al., 2012; Manai et al., 2014) and in cotton (Dong et al., 2014). NO can act (i) as a direct scavenger of ROS, and (ii) antioxidant system inducer to enhance the expression of antioxidant enzyme-encoding genes (Groß et al., 2013). NO applied exogenously may also induce the synthesis of endogenous NO (Hao et al., 2008; Xiong et al., 2009; Zhao et al., 2009; Xu et al., 2010; Fan and Liu, 2012), which can function as signaling molecule or ROS scavenger under prolonged stress conditions by regulating/enhancing the activities of antioxidant enzymes (Hao et al., 2008; Xu et al., 2010; Fan and Liu, 2012) (**Figure 5**).

Consistent with the accumulation of antioxidant enzymes in chickpea plants under salt stress, either alone or co-applied with NO (**Figure 3**), the expression of representative *SOD*, *CAT* and *APX* genes examined was also up-regulated in treated chickpea plants (**Figure 4**). This result suggested that up-regulation of *SOD*, *CAT* and *APX* genes might enhance activities of the SOD, CAT and APX enzymes, thereby providing better protection to the cells against oxidative stress triggered by high salinity (Lu et al., 2007; Yamane et al., 2010; Hu et al., 2012). In support of our finding, several studies also reported the up-regulation of antioxidant enzyme-encoding genes under stress with or without NO treatment. For instance, Hernandez et al. (2000) have reported the enhanced expression of *SOD* and *APX* genes in a NaCl-tolerant *Pisum sativum* variety in comparison with the sensitive variety. Up-regulation of *SOD* gene expression has also been reported in other plants, including *Lycopersicon esculentum* (Aydin et al., 2014) and *Lotus japonicus* (Rubio et al., 2009) under NaCl stress. Zhang et al. (2014) showed the up-regulation of *CAT* and *APX* genes in *Limonium sinense* under high salinity. Menezes-Benavente et al. (2004) and Shafi et al. (2015) reported enhanced expression of *APX* gene in rice and *Arabidopsis*, respectively, under salt stress. Lin et al. (2011) noticed induced expression of *APX* by exogenous NO supply in sweet potato (*Ipomoea batatas*) under wounding stress. Therefore, it is reasonable to conclude that NO may activate the expression of antioxidant enzymes-related biosynthetic genes, which leads to accumulation of antioxidant enzymes, thereby providing better tolerance to plants under stresses (**Figure 5**).

## Conclusion

Our study demonstrates that exogenous supply of NO is effective in mitigating salt stress in chickpea plants. Therefore, application of exogenous NO or manipulation of endogenous NO content might be promising approach for salt stress management in the era of climatic changes.

## Author Contributions

PA and AA designed the experimental work. PA, AH, and EA performed the experimental work. SG carried out the statistical analysis and formatting of the manuscript. PA, AA, and L-ST wrote and revised the manuscript.

## Conflict of Interest Statement

The authors declare that the research was conducted in the absence of any commercial or financial relationships that could be construed as a potential conflict of interest.
